# Supplementary material for: Multiple Myeloma Relapse Is Associated with Increased NFκB Pathway Activity and Upregulation of the Pro-Survival BCL-2 Protein BFL-1
Source: Cancers (Basel). 2021 Sep 17;13(18):4668. doi: 10.3390/cancers13184668 (PMC8467450; doi:10.3390/cancers13184668)
Supplement: Supplementary file 1 [file cancers-13-04668-s001.zip › cancers-1331849-supplementary.pdf]

Supplementary Material: Multiple Myeloma Relapse Is associated with Increased NFκB Pathway Activity and Upregulation of the Pro-Survival BCL-2 Protein BFL-1

Ingrid Spaan, Anja van de Stolpe, Reinier A. Raymakers and Victor Peperzak

Table S1. Matrix showing statistical significant differences in NFκB activity score as analyzed by signal transduction pathway activity (STA) analysis in plasma cells (PC) from newly diagnosed MM patients, stratified by molecular clusters, as incorporated in dataset GSE19784. ns, not significant, **P* <.05, ***P* <.01, ****P* <.001, *****P* <.0001.

	CD-1	CD-2	MF	MS	HY	PR	NFκB	CTA
CD-1		ns	ns	ns	ns	ns	**	ns
CD-2			ns	ns	ns	ns	ns	ns
MF				ns	ns	ns	****	ns
MS					ns	ns	***	ns
HY						ns	*	ns
PR							**	ns
NFκB								ns
CTA								

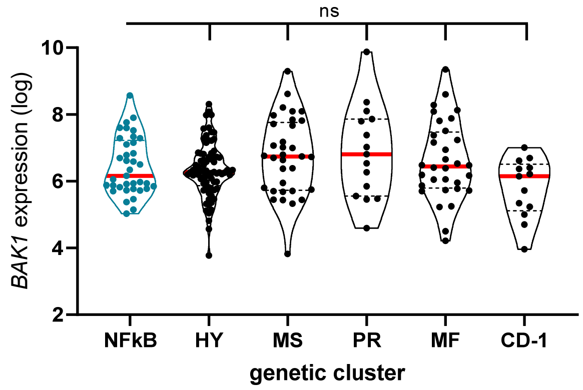

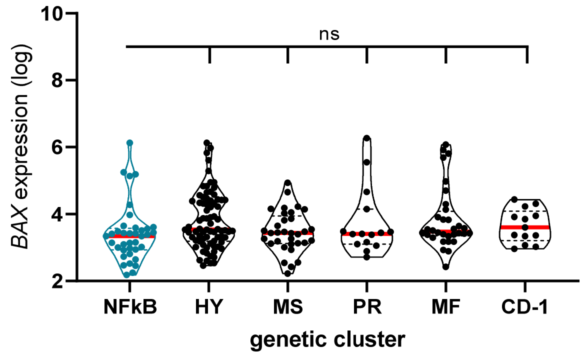

Figure S1. Violin plots showing *BAK1* and *BAX* mRNA transcript expression in PC of newly diagnosed MM patients, as incorporated in dataset GSE19784, in molecular cluster NFκB, and molecular clusters HY, MS, PR, MF, and CD-1 that all showed a significant lower STA NFκB activity score compared to molecular cluster NFκB in Figure 1C. Included samples are represented by individual datapoints, solid red lines indicate the median, and dashed lines the quartiles of the population. ns, not significant. .
